# Novel magnetic resonance imaging methodology for dynamic visualization of respiratory thoracic motion: a pilot feasibility study

**DOI:** 10.3389/fresc.2025.1540183

**Published:** 2025-08-12

**Authors:** Masaaki Kobayashi, Hideyuki Fukubayashi, Katsuma Iwai, Kazumo Miura, Akio Yamamoto, Kumiko Ono, Akinori Miki, Takashi Ueguchi, Akira Ishikawa

**Affiliations:** ^1^ Kobe Co-Medical College, Kobe, Japan; ^2^ Kobe University Graduate School of Health Sciences, Kobe, Japan; ^3^ Center for Information and Neural Networks, Advanced ICT Research Institute, National Institute of Information and Communications Technology, Suita, Japan; ^4^ Department of Radiation Oncology, Osaka University Graduate School of Medicine, Suita, Japan; ^5^ Department of Medical Imaging Science, Osaka University Graduate School of Medicine, Suita, Japan

**Keywords:** magnetic resonance imaging, thoracic movement, dynamic imaging, semi-prone position, respiratory mechanics, positional therapy, pilot study

## Abstract

**Introduction:**

Positional management is important in respiratory rehabilitation. Current magnetic resonance (MR) imaging techniques for visualizing respiratory mechanics are limited by external pressure from receiver coils or spatial restrictions within the bore, and there is no established method for visualizing respiratory movements in the semi-prone position. Therefore, we aimed to develop a novel MR imaging and analysis method for visualizing thoracic movements during free breathing, enabling assessment of positional effects.

**Methods:**

Five healthy male participants were enrolled. MR images were obtained in the supine and semi-prone positions using a fast imaging sequence, allowing for continuous dynamic imaging during deep breathing. Subsequently, an image processing pipeline was applied to enhance visibility. The thoracic expansion was measured and compared between the two positions. Intra- and interobserver reproducibility and test-retest reproducibility were assessed using intraclass correlation coefficients (ICCs).

**Results:**

The proposed method enabled successful dynamic visualization of thoracic movements without using a receiver coil. A significant difference in thoracic expansion between the supine and semi-prone positions was observed in the head-foot and right-left directions. Head-foot expansion was greater in the supine position, while right-left expansion was greater in the semi-prone position. No significant differences were found in the anterior-posterior direction. Both intra- and interobserver reproducibility were high, with ICCs exceeding 0.9 for most thoracic expansion measurements. Test-retest reproducibility also demonstrated high agreement for most measurements, with ICCs ranging from 0.74 to 0.97 across different directions and positions.

**Conclusion:**

The developed MR imaging method allows for noninvasive visualization of thoracic movements during natural breathing with robust reproducibility. This method could provide valuable insights into respiratory mechanics, supporting its clinical application in respiratory rehabilitation.

## 1 Introduction

In respiratory rehabilitation, positional changes are implemented to provide breathing assistance and facilitate sputum clearance. The importance of positional management in respiratory rehabilitation has gained increasing recognition, particularly in light of the coronavirus disease 2019 pandemic (1–3). Prone positioning augments ventilation in the dorsal lung regions and improves oxygenation, thereby improving the survival outcomes of patients with severe respiratory failure (4). However, prone therapy imposes substantial burdens on both healthcare providers and patients, leading to low implementation rates (5, 6).

The semi-prone position, an intermediate posture between the prone and lateral decubitus positions, serves as an alternative to prone positioning (7–9). Semi-prone positioning is useful in respiratory rehabilitation, facilitating positional management, sputum clearance, and aspiration prevention (10–13). Due to its ease of implementation and reduced burden on both patients and healthcare staff, the semi-prone position is more readily adopted in clinical settings (14). The unique distribution of gravitational forces in this position may result in distinct respiratory movements compared with those observed in the supine or prone positions. Visualizing and understanding the respiratory mechanics in the semi-prone position could provide valuable insights for healthcare professionals involved in positional management and respiratory care.

Various imaging modalities are available for assessing respiratory function, including computed tomography, ultrasonography, magnetic resonance (MR) imaging, and electrical impedance tomography (EIT). However, computed tomography involves radiation exposure, making it unsuitable for studies with healthy volunteers; ultrasonography cannot visualize the entire thorax (15), limiting its utility for comprehensive assessment of thoracic motion; and EIT visualizes regional ventilation rather than anatomical movements, limiting its role in evaluating thoracic motion (16). Conversely, MR imaging offers a noninvasive approach for dynamically assessing diaphragmatic and chest wall movements during respiration without radiation exposure (17–19).

Using MR imaging, Kondo et al. demonstrated a correlation between thoracic expansion and lung volume in the supine position; however, they did not investigate other positions (20). Mase et al. documented differences in regional lung volumes across various postures, highlighting that semi-prone positioning reduces cardiac compression on the lungs (21). Nevertheless, since these studies involved 30 s breath-holding during imaging, the images may not truly represent physiological respiratory states. Furthermore, information on MR imaging techniques and analysis methods remains limited, particularly regarding essential factors such as the selection of receiver coils used for signal reception, which is crucial for reproducing experiments and interpreting results.

All clinical MR scanners are equipped with a “body coil”, which is integrated into the gantry. This coil is typically used to transmit radiofrequency electromagnetic waves into the body, and can also receive the MR signals induced in response from the body. However, due to its relatively low signal sensitivity, it is rarely used for signal reception in clinical imaging. For thoracic imaging, a dedicated phased-array receiver coil (often called a “body-array coil”) is commonly used instead to achieve high signal-to-noise ratio (SNR) for signal reception (22). This type of coil is typically placed around the thorax, covering both the anterior and posterior sides. In this study, however, we intentionally employed the built-in body coil for signal reception instead of the phased-array receiver coil, for two reasons. First, the phased-array receiver coil may compress the chest due to its weight. Second, in the semi-prone position, proximity to the MR scanner's bore can cause interference with the coil, complicating patient insertion into the bore or causing significant coil-induced chest compression once inserted. While using such coils may lead to deviations from the physiological respiratory state, omitting them results in inadequate signal acquisition and low-SNR images.

Currently, there is no established method for visualizing respiratory movements in the semi-prone position; consequently, available data on the respiratory mechanics in this position are extremely limited. Therefore, we aimed to develop an MR imaging and analysis method that enables visualization of thoracic movements during free breathing in the semi-prone position without any coil-induced loading or spatial constraints within the bore. The goal at this current stage is to demonstrate the technical feasibility of this approach and establish preliminary visualization capabilities, rather than to provide precise quantitative measurements.

## 2 Materials and methods

### 2.1 Study design and participants

The study was approved by the institutional review board at Kobe University Graduate School of Health Sciences (approval No.1167). All participants provided written informed consent before participation. All procedures adhered to the tenets of the Declaration of Helsinki. The study is reported following the Strengthening the Reporting of Observational Studies in Epidemiology guidelines (23).

This study, although cross-sectional, was primarily a pilot investigation involving five healthy young adult male volunteers. Participants were recruited at Kobe University Graduate School of Health Sciences between July 2023 and March 2024. MR imaging was conducted at the imaging facilities of Kobe Co-Medical College. The inclusion criteria were males aged 20–40 years, no history of smoking, and no contraindications for MR imaging. We included only male participants because the study aimed to test a novel MR imaging technique without using any phased-array receiver coils, and men generally exhibit greater thoracic motion and have less subcutaneous fat, allowing respiratory motion to be observed more easily than in women. The exclusion criterion was a history of respiratory disease or musculoskeletal disorders that might impair posture maintenance.

Prior to imaging, all participants underwent a series of baseline assessments. We measured height and weight using a standard stadiometer and scale and calculated the body mass index. Physical activity levels were assessed using the short form of the International Physical Activity Questionnaire (24). Pulmonary function tests were conducted using a Minato AS-307 spirometer (Minato Medical Science Co., Ltd., Osaka, Japan). We measured forced vital capacity (FVC), %FVC, forced expiratory volume in 1 s (FEV_1_), %FEV_1_, and FEV_1_/FVC. Reference values were calculated based on the LMS method (2014) as outlined by the Japanese Respiratory Society (25).

### 2.2 MR image acquisition

MR images were obtained using a 1.5-T Vantage Elan MRT-2020 system (Canon Medical Systems Corporation, Otawara, Japan). Signal transmission and reception were achieved using a built-in body coil within the gantry.

**Figure 1:**
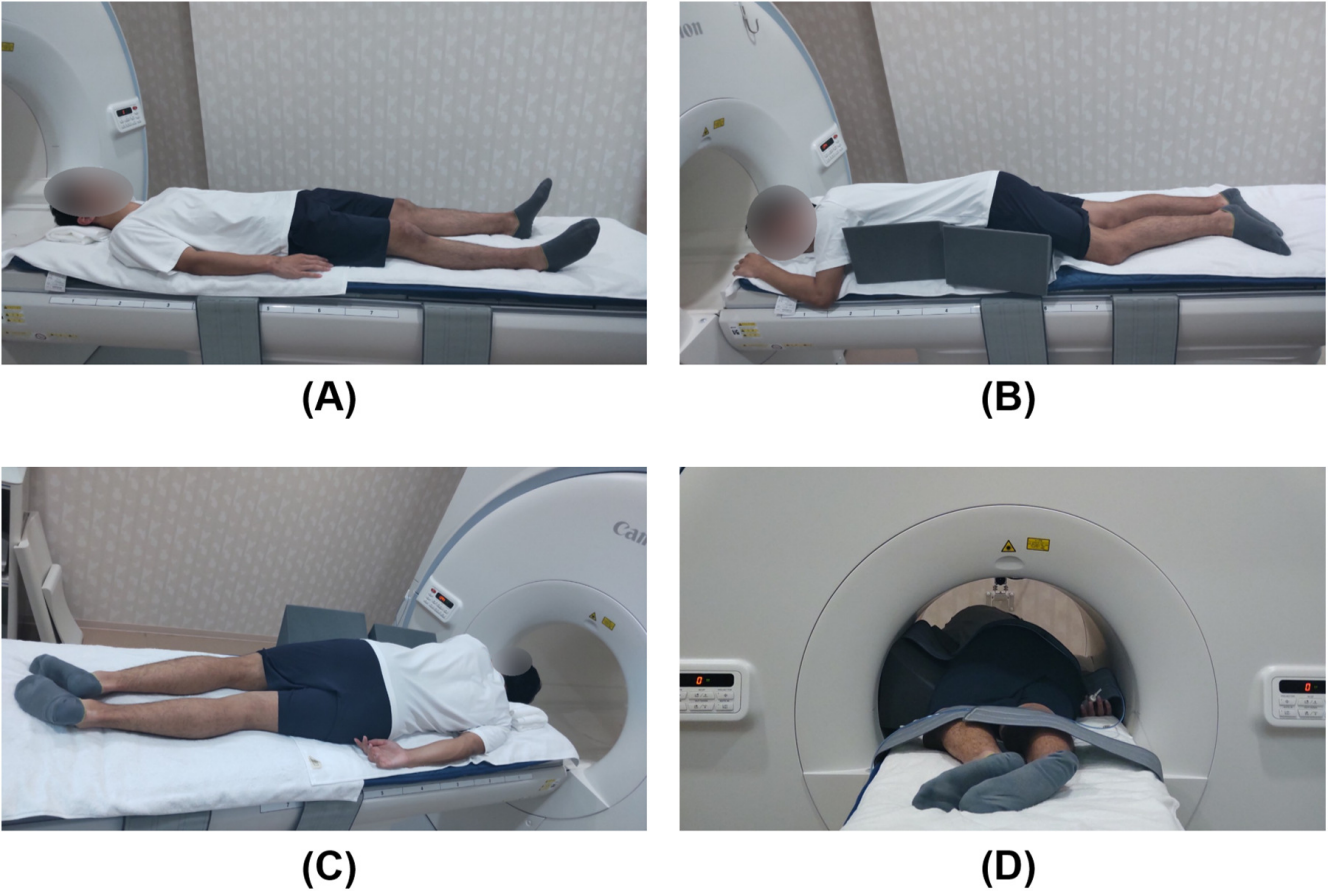
Participant positioning for magnetic resonance imaging. A sleeping mat and towels were placed on the examination table to enhance participants' comfort. **(A)** Supine position; **(B)** Semi-prone position with the right side down, supported by a triangular positioning block with an inclination set to 35 degrees to prevent torsion between the pelvic and shoulder girdles; **(C)** Semi-prone position viewed from the dorsal side; **(D)** The participant is inserted into the bore in the semi-prone position. There is sufficient space between the participant and the bore, indicating no interference between them.

To mitigate potential bias, participants were instructed to refrain from consuming food and beverages, with the exception of water, for a minimum of 3 h before the imaging and were attired in standard examination gowns. A 5-cm-thick mat and towels were positioned on the examination table beneath each participant. Participants were gently restrained with loosely wrapped belts set while in a state of deep inspiration to prevent respiratory-induced compression from the belts. Before imaging, they were instructed to remain motionless and follow specific breathing instructions. Images were initially acquired in the supine position, subsequently followed by the semi-prone position with the right side down. For the semi-prone position, an MR-compatible triangular positioning block with a 35-degree inclination was utilized to maintain a uniform torso angle (Figure 1).

All imaging procedures were conducted by an experienced radiological technologist. Images were acquired in two planes, set using three-directional localizer images: (i) coronal sections parallel to the torso passing through the tracheal bifurcation, and (ii) sagittal sections parallel to the spine passing through the midpoint of the diaphragm in both the right and left hemithoracic cavities. A two-dimensional (2D) fast field echo sequence was employed with the following imaging parameters: repetition time, 3.9 ms; echo time, 1.3 ms; flip angle, 5°; matrix size, 192 × 192; field of view, 500 × 500 mm; and slice thickness, 8.0 mm. This provided a temporal resolution of 0.6 s, allowing for continuous dynamic imaging over a 1-min period by repeating this sequence. Participants performed deep breathing for 60 s during the acquisition.

To assess test-retest reproducibility, a second imaging session was conducted approximately one week after the first, following the same procedures.

### 2.3 MR image analysis

Image analysis was performed using the open-source image processing software ImageJ (version 1.54b; National Institutes of Health, Bethesda, MA, USA). The analysis workflow consisted of two stages: automated preprocessing and subsequent manual evaluation, as detailed below. Within the analysis workflow, the automated preprocessing represents the core technical contribution of this study, aimed at establishing the feasibility of thoracic motion visualization, while the manual evaluation demonstrates that basic quantitative measurements can be obtained using this approach.

#### 2.3.1 Automated preprocessing

**Figure 2:**
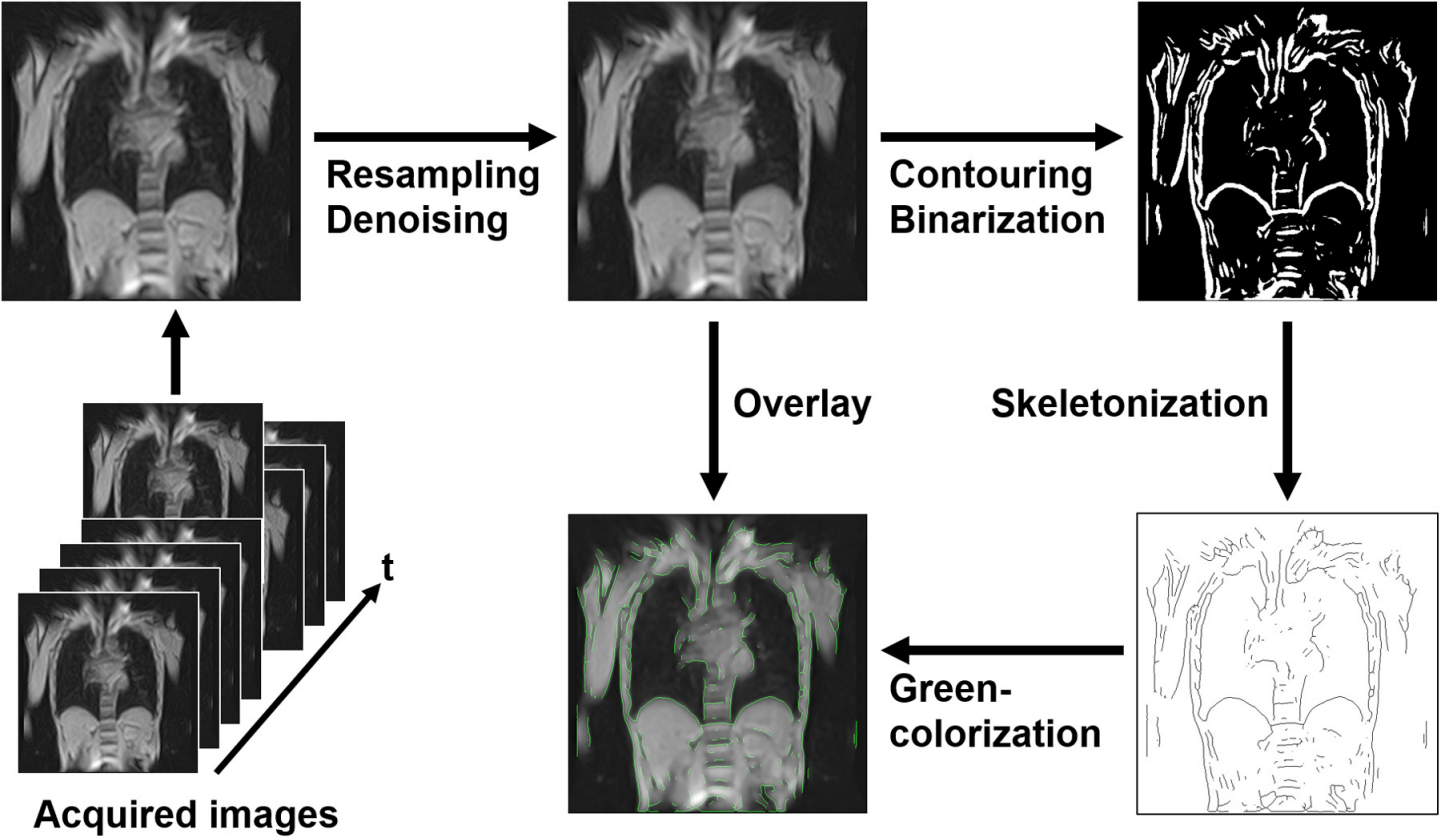
Preprocessing pipeline for magnetic resonance image analysis. Initially, the acquired images were resampled and denoised. Next, contouring and binarization were performed, followed by skeletonization to reduce contours to a single-pixel width. The processed contours were colorized in green and overlaid onto the denoised images to enhance thoracic contours. This automated preprocessing pipeline facilitated the analysis of thoracic expansion through dynamic imaging.

Initially, all raw images were interpolated to a matrix size of 512 × 512 to enhance apparent spatial resolution, followed by denoising using a 3 × 3 median filter. Edge detection was then performed using horizontal and vertical spatial derivatives via the Sobel filter, followed by root mean square calculations. The resulting contour images were binarized and further processed by skeletonization to reduce contour components to a single-pixel width. These processed contours were colorized and overlaid onto the denoised images to produce time-series images with enhanced thoracic contours (Figure 2). All preprocessing steps were implemented using standard ImageJ functions with default parameters and automated via its macro functionality.

#### 2.3.2 Manual evaluation

##### 2.3.2.1 Generation of datasets

**Figure 3:**
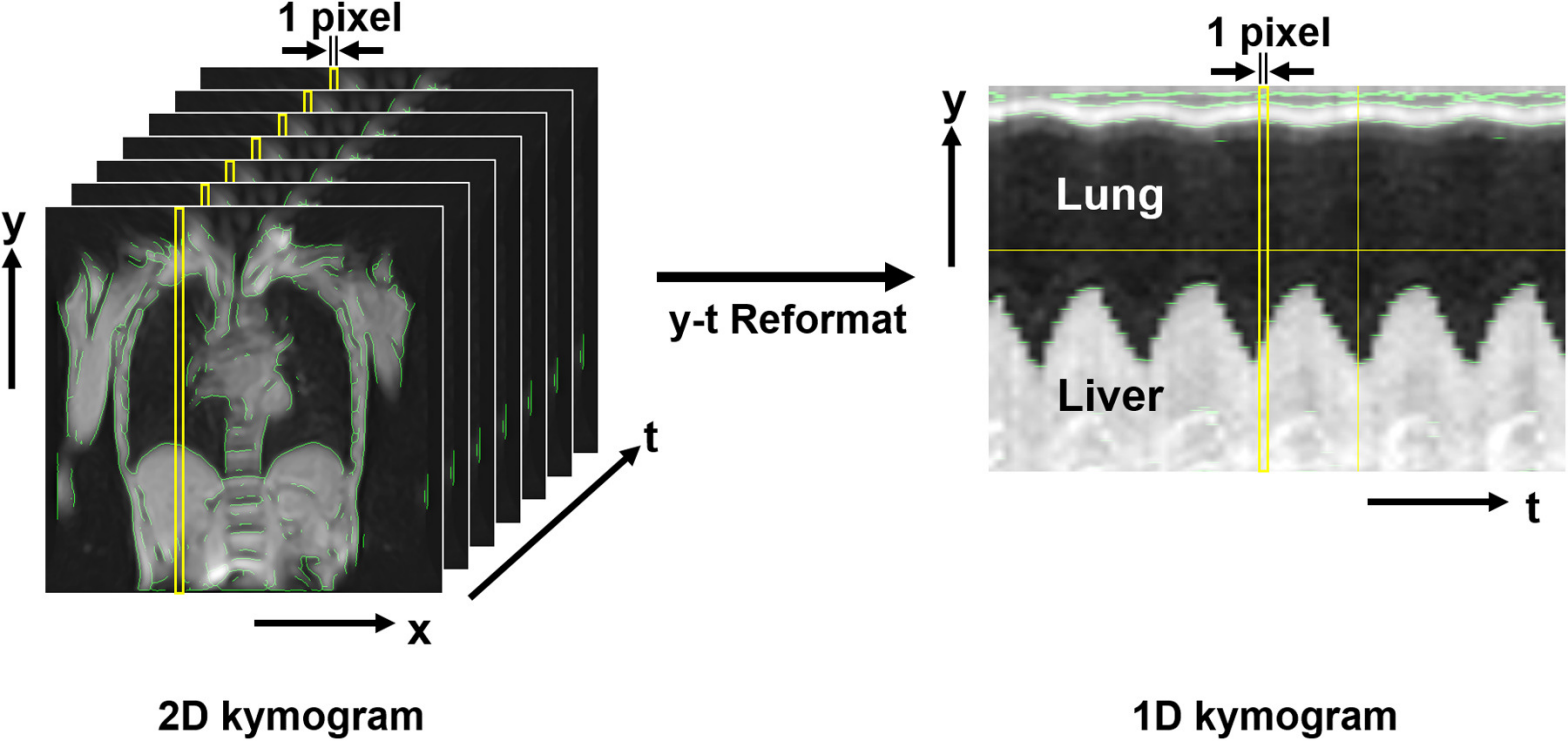
Magnetic resonance-based kymogram—a time-space visualization of the temporal dynamics of respiratory motion. The figure presents the method for visualizing the temporal dynamics of respiratory motion, named magnetic resonance (MR)-based “kymogram”. The 2D kymogram is a temporal series of 2D dynamic MR images, depicting 2D respiratory thoracic motion. From this 2D kymogram, 1D kymograms can be reconstructed by extracting pixel information (i.e., signal intensities) in the y-direction at any given x-coordinate across all time points (1D y-t kymogram) or by extracting pixel information in the x-direction at any given y-coordinate across all time points (1D x-t kymogram). This figure shows an example of a 1D y-t kymogram that captures the movement of the lung and liver during different respiratory phases. The 1D kymogram is produced with a resolution of 1 pixel along the t-axis, allowing for visualization of thoracic movements along the spatial and temporal axes.

**Figure 4:**
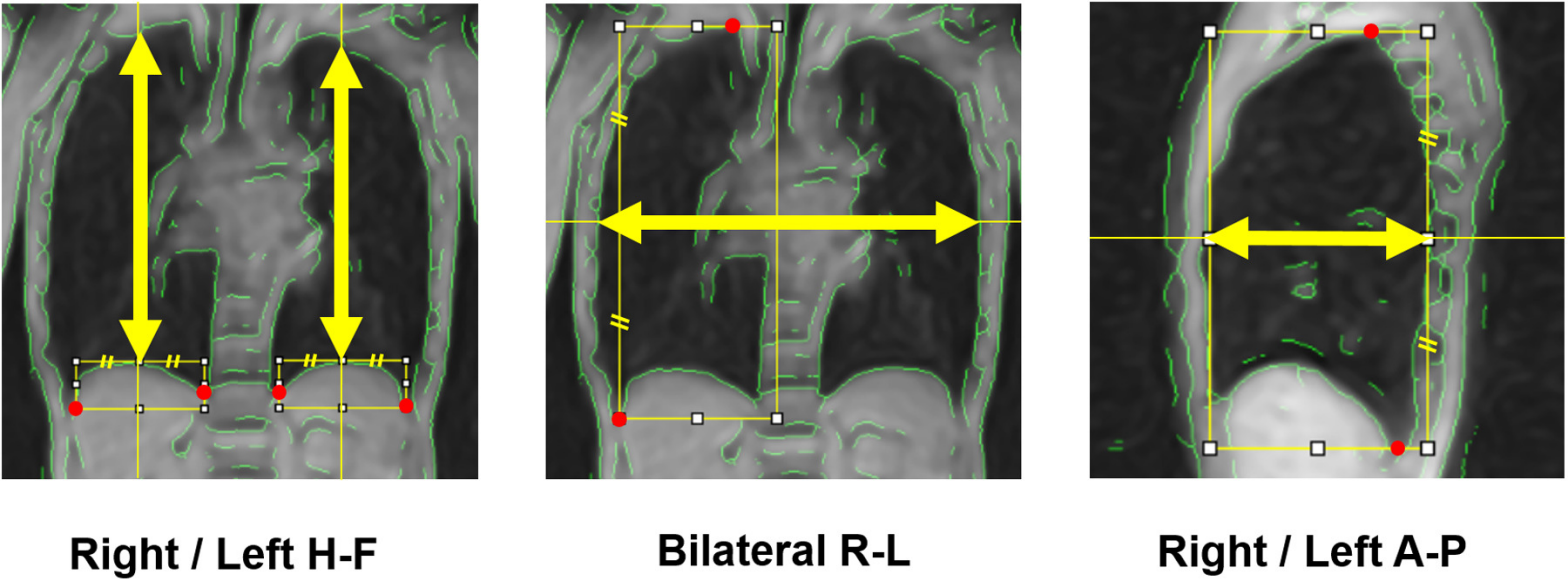
Measurement axes of respiratory thoracic expansion. The respiratory thoracic expansion was measured in the head-foot (H-F) and anterior-posterior (A-P) directions in both hemithoraces and in the bilateral right-left (R-L) direction, each along the axis indicated by yellow arrows. To determine these measurement axes, visible anatomical landmarks (red marks) were first identified, then auxiliary lines were drawn, and finally midpoints were calculated, as illustrated in the figure.

The resulting data constituted three-dimensional images (2D kymograms) with horizontal (x), vertical (y), and temporal (t) axes. By placing a crosshair cursor at an arbitrary spatial position (x, y), ImageJ can extract their x-t and y-t components passing through that point, thereby generating 1D kymograms to visualize temporal changes in thoracic contours across respiratory phases (Figure 3). Thoracic expansion was measured on 1D kymograms obtained at five locations, as shown in Figure 4: along the head-foot (H-F) directions of the right and left hemithoraces, the bilateral right-left (R-L) direction, and the anterior-posterior (A-P) directions of the right and left hemithoraces. For the H-F directions, the measurement planes were set to pass through the midpoints of the visible right and left hemidiaphragms on coronal 2D kymograms. For the bilateral R-L direction, the measurement plane was set at a height corresponding to the midpoint between the lateral inferior edge of the right hemidiaphragm and the uppermost point of the right hemithorax on coronal 2D kymograms. For the A-P directions, the measurement planes were set to pass through the midpoint between the dorsalmost inferior edge of the right hemidiaphragm and the uppermost point of the right thoracic cavity. In all cases, the midpoints were obtained by manually identifying two visible anatomical landmarks on the 2D kymograms and calculating the midpoint of their coordinates along the respective axes, as illustrated with auxiliary lines in Figure 4. Although the 1D kymograms typically contained 6–10 respiratory cycles during the 60 s acquisition, cycles that were clearly inconsistent compared with other cycles in the pattern of lung motion were visually identified and excluded from the subsequent analysis to ensure consistency. All operations were performed by a single observer (M.K.).

##### 2.3.2.2 Measurement of respiratory thoracic expansion

**Figure 5:**
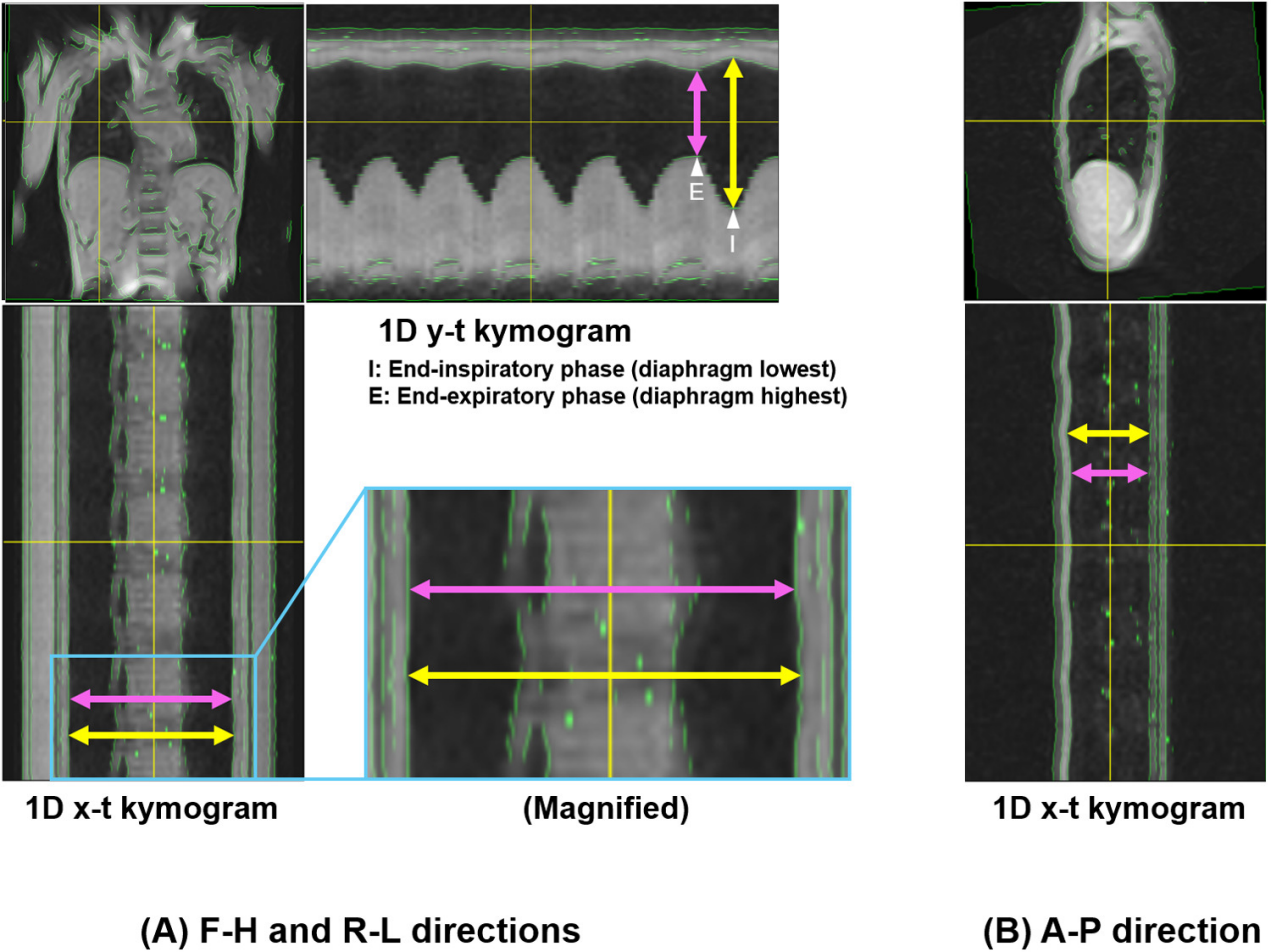
Measurement of thoracic expansion on 1D kymograms. Measurements of thoracic dimensions were performed at single time points of end-inspiratory and end-expiratory phases at which the diaphragm reached its lowest and highest positions throughout the entire 60 s duration on 1D y-t kymograms. **(A)** 1D y-t kymogram showing measurements of H-F thoracic dimensions, and 1D x-t kymogram showing R-L dimensions. The end-inspiratory and end-expiratory phases for the measurements are also shown on the y-t kymogram (arrowhead with “I” and that with “E”, respectively). The magnified image shows slight thoracic expansion in the R-L direction. **(B)** 1D x-t kymogram showing measurements of A-P dimensions. Yellow and pink arrows correspond to the measurements of thoracic dimensions at the end-inspiratory and end-expiratory phases, respectively. Note that in this representative example, the selected time points happened to occur within the same respiratory cycle, but were not necessarily from the same cycle.

Measurements were conducted on the 1D kymograms as described above. Given the temporal resolution limitations (0.6 s) and the inherently reduced spatial resolution of body coil imaging, precise identification of individual end-expiratory and end-inspiratory coordinates at discrete time points was found challenging with the current analysis pipeline, even with the assistance of green contour overlays, which is one of the limitations discussed later. Therefore, at this stage, we visually identified the end-inspiratory and end-expiratory phases using the following approach, and then measured thoracic dimensions to calculate thoracic respiratory expansion. Of the two types of 1D kymograms (x-t and y-t kymograms) generated from each of coronal and sagittal 2D kymograms, only the y-t kymograms can depict respiratory diaphragmatic movement. Thus, on the y-t kymograms with the assistance of ImageJ cross-hair cursor and measurement functions, we visually identified the single time points of end-inspiratory and end-expiratory phases at which the diaphragm reached its lowest and highest positions throughout the entire 60 s acquisition duration, regardless of whether they occurred within the same respiratory cycle or different cycles. For measurements in the H-F direction, thoracic dimensions were measured as the distance between the upper and lower contours of the lung on the y-t kymograms at these time points (Figure 5A). For measurements in the R-L direction, thoracic dimensions were measured on the x-t kymograms as the distance between the right contour of the right lung and the left contour of the left lung at the same time points determined on the corresponding y-t kymograms (Figure 5A). For the measurements in the A-P direction, thoracic dimensions were measured on the x-t kymograms as the distance between the anterior and posterior contours of the lung (Figure 5B) at the end-inspiratory and expiratory time points determined on the corresponding y-t kymograms. The measurements were performed strictly orthogonal to the temporal axis on the respective 1D kymograms using the graphical measurement tools available in ImageJ assisted by the overlaid green contour lines to facilitate the identification of thoracic boundaries.

### 2.4 Measurement reproducibility

To evaluate the reproducibility of thoracic expansion measurements obtained using the proposed method, we assessed intraobserver, interobserver, and test-retest agreement using intraclass correlation coefficients (ICCs). Two observers (M.K. and T.U.) with extensive experience in thoracic MR imaging and respiratory physiology participated in this section. Intraobserver reproducibility was assessed by having Observer 1 (M.K.) analyze the data from the first imaging session twice, with a one-week interval between the two analysis sessions. ICCs were calculated based on the thoracic expansion values obtained from these two independent analyses. Interobserver reproducibility was assessed by having Observer 2 (T.U.) independently analyze the same data from the first imaging session, without any reference to the results of Observer 1. ICCs were calculated by comparing the measurements obtained by Observer 2 with those from the first analysis session conducted by Observer 1. Test–retest reproducibility was evaluated to account for the entire imaging process, including participant setup, image acquisition, and analysis. For this purpose, Observer 1 analyzed the data from the second imaging session, and the results were compared to those from the first analysis of the first imaging session. ICCs were calculated to assess agreement between these two time points.

### 2.5 Statistical analysis

Thoracic expansion measurements were compared between the supine and semi-prone positions using the Wilcoxon signed-rank test, based on two sets of data obtained by Observer 1: the first analysis of the first imaging session and the analysis of the second imaging session. *P*-values were adjusted for multiple comparisons using the Benjamini–Hochberg method. All statistical analyses were conducted using R software (version 4.3.1; R Core Team, Vienna, Austria), with the significance level set at 5%. In addition, 95% confidence intervals were calculated for all ICC values. ICCs were interpreted as follows: 0.00–0.20, slight agreement; 0.21–0.40, fair agreement; 0.41–0.60, moderate agreement; 0.61–0.80, substantial agreement; and 0.81–1.00, near-perfect agreement (26).

## 3 Results

### 3.1 Participants' characteristics

The study participants had a mean age of 29.8 years and a mean body mass index of 24.5 kg/m^2^. Pulmonary function test results were all within normal ranges (Table 1).

**Table 1:** Participant characteristics.

Characteristic	Value
Age (years)	29.8 ± 7.2
Height (cm)	169.2 ± 5.4
Weight (kg)	69.8 ± 8.1
BMI (kg/m^2^)	24.5 ± 3.5
IPAQ level*	2.0 ± 1.0
FVC (L)	4.5 ± 0.7
%FVC (%)	100.0 ± 9.0
FEV_1_/FVC (%)	82.6 ± 8.4
FEV_1_ (L)	3.7 ± 0.5
%FEV_1_ (L)	96.5 ± 10.4

Data are presented as mean ± standard deviation. BMI, body mass index; IPAQ, international physical activity questionnaire—Short Form [* 1 = low physical activity; 2 = moderate physical activity; 3 = high physical activity]; FVC, forced vital capacity; %FVC, percentage of forced vital capacity; FEV_1_/FVC, ratio of forced expiratory volume in 1 s to forced vital capacity; FEV_1_, forced expiratory volume in 1 s; %FEV_1_, percentage of forced expiratory volume in 1 s.

### 3.2 Image characteristics

**Figure 6:**
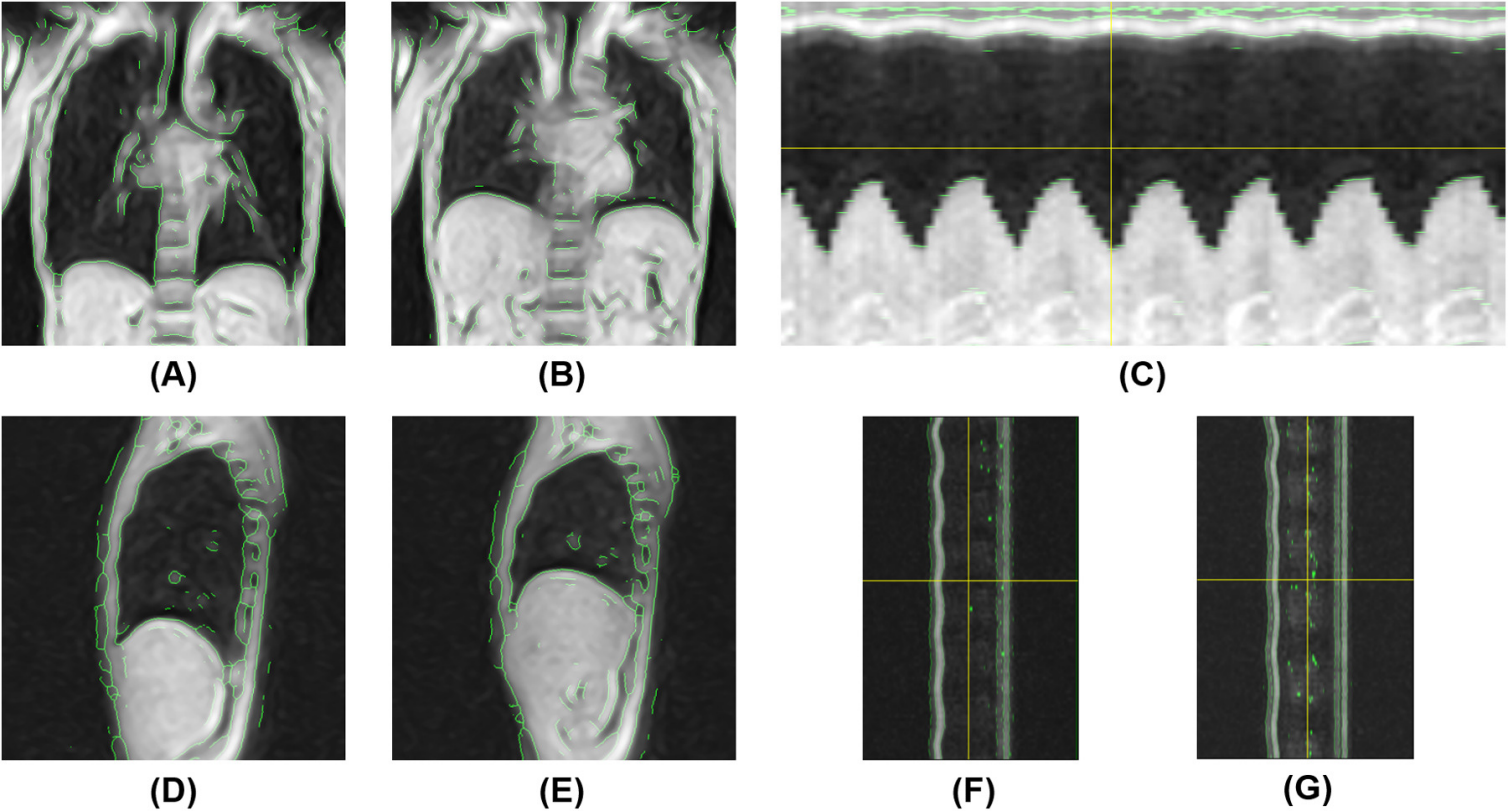
Magnetic resonance-based visualization of thoracic respiratory motion in the supine position. **(A)** Coronal 2D kymogram at the end of inspiration; **(B)** Coronal 2D kymogram at the end of expiration; **(C)** 1D kymogram of the right hemithorax; **(D)** Sagittal 2D kymogram of the right hemithorax at the end of inspiration; **(E)** Sagittal 2D kymogram of the right hemithorax at the end of expiration; **(F)** 1D kymogram of the right hemithorax; **(G)** 1D kymogram of the left hemithorax.

**Figure 7:**
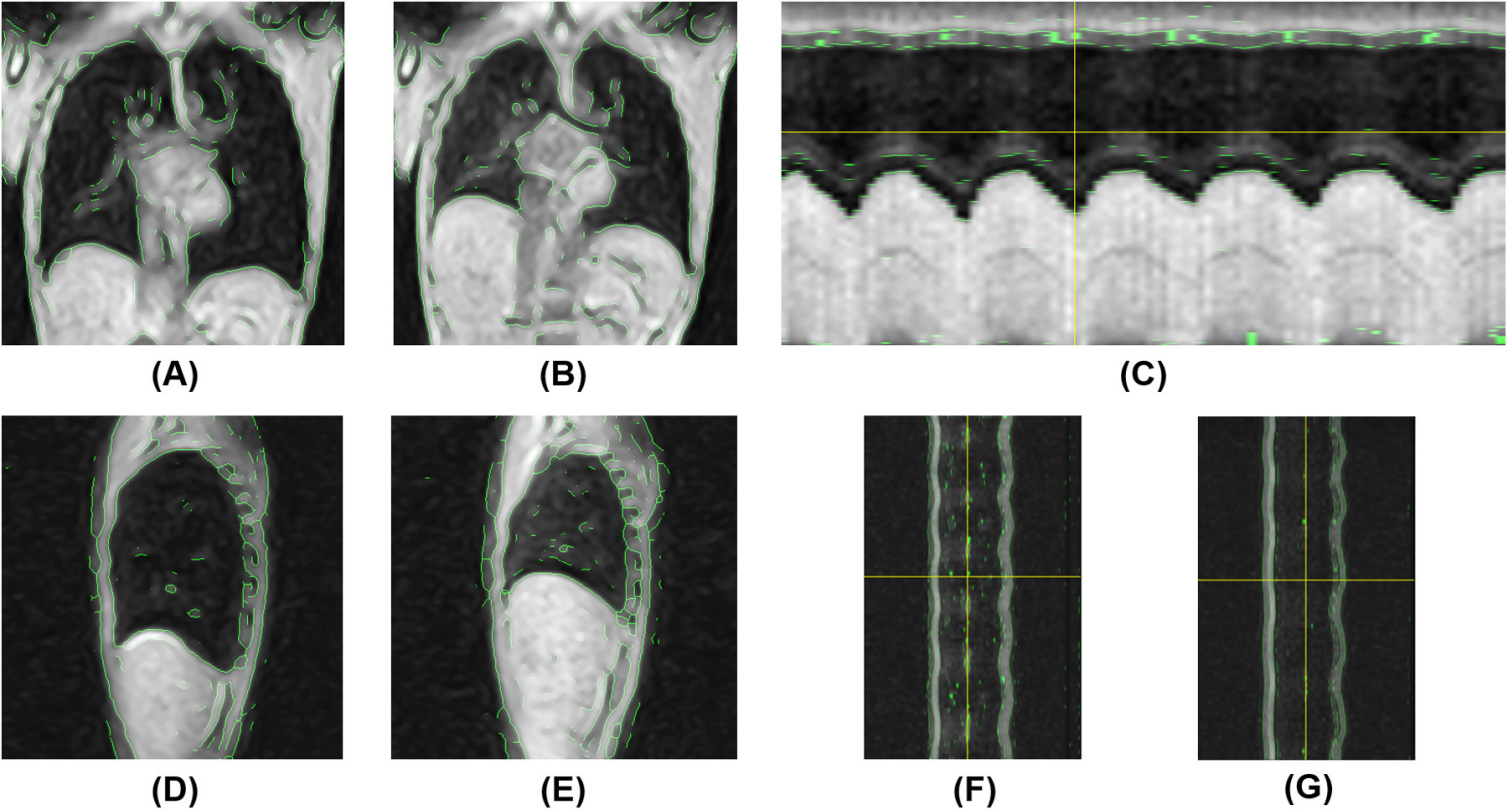
Magnetic resonance-based visualization of thoracic respiratory motion in the semi-prone position. **(A)** Coronal 2D kymogram at the end of inspiration; **(B)** Coronal 2D kymogram at the end of expiration; **(C)** 1D kymogram of the right hemithorax; **(D)** Sagittal 2D kymogram of the right hemithorax at the end of inspiration; **(E)** Sagittal 2D kymogram of the right hemithorax at the end of expiration; **(F)** 1D kymogram of the right hemithorax; **(G)** 1D kymogram of the left hemithorax.

Dynamic thoracic images were successfully acquired in all five participants. In both positions, chest wall contours were clearly recognizable both on coronal and sagittal 2D kymograms, owing to the appropriate image preprocessing. The 1D kymograms effectively visualized the respiratory-induced changes in thoracic shape, with y-t kymograms enabling the identification of end-inspiratory and end-expiratory phases suitable for image analysis. Representative images of the respiratory dynamics in the supine and semi-prone positions are shown in Figures 6, 7, respectively. Continuous respiratory dynamics, including intermediate respiratory phases, can be observed in Supplementary Videos S1–S4.

### 3.3 Comparison of thoracic expansion between the supine and semi-prone positions

In both positions, thoracic expansion was greater in the H-F direction than in the A-P and R-L directions (Table 2). Thoracic expansion significantly differed between the supine and semi-prone positions in the H-F and R-L directions. Right thoracic H-F expansion in the supine position exceeded those in the semi-prone position by approximately 20 mm (*p* = 0.029). The left thoracic H-F expansion in the supine position was approximately 10 mm larger than in the semi-prone position, and this difference was also statistically significant (*p* = 0.036). In the A-P direction, the expansion of the right and left hemithoraces was nearly the same in the supine position, whereas, in the semi-prone position, the right hemithorax (loaded side) expansion was smaller than that of the left hemithorax (non-loaded side), although these differences were not statistically significant. In the bilateral R-L direction, thoracic expansion was slightly greater in the semi-prone position than in the supine position, and this difference reached statistical significance (*p* = 0.036).

**Table 2:** Thoracic dimensions at end-inspiration and end-expiration, and their expansion in the supine and semi-prone positions.

Measurement axes	Supine			Semi-prone			Expansion *p*-value
	End-insp (mm)	End-exp (mm)	Expansion (mm)	End-insp (mm)	End-exp (mm)	Expansion (mm)	
Right H-F	216 ± 20	131 ± 21	84 ± 20	191 ± 23	125 ± 23	66 ± 20	0.029
Left H-F	220 ± 13	138 ± 16	81 ± 18	230 ± 14	161 ± 18	69 ± 16	0.036
Bilateral R-L	261 ± 13	257 ± 13	4 ± 1	259 ± 17	251 ± 14	8 ± 5	0.036
Right A-P	140 ± 17	126 ± 13	14 ± 10	135 ± 18	126 ± 16	9 ± 4	0.063
Left A-P	140 ± 9	126 ± 10	14 ± 10	143 ± 14	127 ± 13	16 ± 10	0.205

Data are presented as mean ± standard deviation. *P*-values were obtained using the Wilcoxon signed-rank test (paired, two-sided) to compare thoracic expansion between the supine and semi-prone positions, adjusted for multiple comparisons by the Benjamini–Hochberg procedure. H-F, head–foot; R-L, right–left; A-P, anterior–posterior; End-Insp, end-inspiration; End-Exp, end-expiration. Expansion = End-Insp—End-Exp.

### 3.4 Measurement reproducibility

In the evaluation of intraobserver reproducibility, overall high agreement was noted between the two independent analyses using the data from the first imaging session for both positions (Table 3). ICC values exceeded 0.9 for all measurements, except for the R-L expansion in the supine position, which showed moderate agreement. A similar trend was observed for interobserver reproducibility (Table 4), with ICC values again exceeding 0.9 for most measurements, except for the R-L expansion in the supine position, which showed fair agreement. For test-retest reproducibility, many measurements showed high ICC values in the range of 0.8 to above 0.9 (Table 5); however ICCs were generally lower compared with intraobserver reproducibility. In particular, the ICC for bilateral R-L expansion in the supine position was unstable, showing a markedly low value. While the ICCs suggest overall high reproducibility, these results should be interpreted with caution. The extremely small sample size (*n* = 5) may have induced statistical instability, leading to wide confidence intervals with some lower boundaries approaching or including zero, or apparent ceiling effects. In addition, for test-retest reproducibility, potential changes in the physiological state of participants between sessions may have contributed to variability. These ICC values are included as preliminary estimates and are supported by Supplementary Tables S1, S2, which present the detailed measurements across observers and sessions.

**Table 3:** ICCs for intraobserver reproducibility.

Measurement axes	Supine		Semi-prone	
Right H-F	1.00	(0.97, 1.00)	1.00	(0.97, 1.00)
Left H-F	0.99	(0.91, 1.00)	1.00	(0.99, 1.00)
Bilateral R-L	0.61	(−0.28, 0.95)	0.97	(0.77, 1.00)
Right A-P	1.00	(0.98, 1.00)	0.99	(0.90, 1.00)
Left A-P	0.99	(0.96, 1.00)	0.98	(0.85, 1.00)

ICC, intraclass correlation coefficient; H-F, head-foot; R-L, right-left; A-P, anterior-posterior. Numbers in parentheses are 95% confidence intervals (lower limit, upper limit).

**Table 4:** ICCs for interobserver reproducibility.

Measurement axes	Supine		Semi-prone	
Right H-F	1.00	(0.94, 1.00)	1.00	(0.65, 1.00)
Left H-F	1.00	(0.95, 1.00)	1.00	(0.91, 1.00)
Bilateral R-L	0.52	(−0.19, 0.93)	0.90	(0.33, 0.99)
Right A-P	0.99	(0.92, 1.00)	0.94	(0.62, 0.99)
Left A-P	0.99	(0.83, 1.00)	0.98	(0.87, 1.00)

ICC, intraclass correlation coefficient; H-F, head-foot; R-L, right-left; A-P, anterior-posterior. Numbers in parentheses are 95% confidence intervals (lower limit, upper limit).

**Table 5:** ICCs for test-retest reproducibility.

Measurement axes	Supine		Semi-prone	
Right H-F	0.97	(0.79, 1.00)	0.96	(0.75, 1.00)
Left H-F	0.92	(0.54, 0.99)	0.74	(−0.04, 0.97)
Bilateral R-L	−0.47	(−0.91, 0.54)	0.93	(0.58, 0.99)
Right A-P	0.80	(0.11, 0.98)	0.89	(0.42, 0.99)
Left A-P	0.95	(0.66, 0.99)	0.95	(0.70, 0.99)

ICC, intraclass correlation coefficient; H-F, head-foot; R-L, right-left; A-P, anterior-posterior. Numbers in parentheses are 95% confidence intervals (lower limit, upper limit).

## 4 Discussion

In respiratory rehabilitation, appropriate patient positioning is fundamental for providing breathing assistance and facilitating sputum clearance. However, understanding how positional changes affect thoracic respiratory movements remains a critical yet underexplored issue. In this study, we developed a novel MR imaging and analysis method that enabled noninvasive visualization of spatiotemporal changes in thoracic morphology during respiration in both the supine and semi-prone positions. Using this method, we found that respiratory thoracic expansion significantly differs between these positions in the H-F and R-L directions: H-F expansion was greater in the supine position, whereas R-L expansion was slightly greater in the semi-prone position. Additionally, we evaluated the precision of this method from a reproducibility perspective, demonstrating its overall high consistency in intra- and interobserver evaluations, as well as good test-retest agreement when considering potential physiological variability between sessions.

Phased-array receiver coils are essential in clinical MR imaging as they dramatically improve image quality by enhancing the SNR (22). Not using a phased-array coil may substantially compromise the SNR, resulting in noisier and less clear images. However, particularly in the semi-prone position, the body's inclination presents challenges when using a phased-array coil for signal reception, as interference within the bore and the coil's weight may potentially affect respiratory movements. To mitigate these drawbacks, we developed an MR imaging and analysis method that does not require the use of a phased-array coil. One of the greatest technical challenges was how to compensate for the dramatic loss of SNR caused by not using a phased-array coil—in other words, relying solely on the gantry-integrated body coil, which is not typically used for signal reception in clinical imaging. For visualizing thoracic movements, we prioritized obtaining images that could capture the entire thorax at once rather than high-resolution images typically used in diagnostic imaging. Given that the thorax is composed of a set of curves with small curvatures, we hypothesized that lowering the image sampling density, combined with appropriate interpolation between pixels, could enable adequate representation of thoracic morphology. Reducing the sampling density means increasing voxel size during signal acquisition, which can effectively enhance the SNR and reduce acquisition time per image. Moreover, to achieve the necessary temporal resolution for visualizing thoracic movements, we employed a 2D fast field echo sequence, which balances both SNR and temporal resolution. Through image processing techniques, such as interpolation, edge-preserving noise reduction using median filtering, contour detection, and skeletonization, we were able to produce clear visual representations of thoracic contours and obtain 1D and 2D kymograms that clearly depicted thoracic movements.

Regarding thoracic expansion measurements, our results are generally consistent with those of previous studies. In our study, H-F expansion was approximately 80 mm in the supine position and 70 mm in the semi-prone position. Haji et al. showed that diaphragmatic movement reaches 7–10 cm during deep breathing in the supine position (27). Plathow et al. reported similar findings, indicating a diaphragmatic displacement of approximately 6–9 cm (28). In the semi-prone position, H-F expansion tended to be smaller than that in the supine position. This could be attributed to the fact that body weight generates pressure on the anterior thorax and the load-bearing side, restricting thoracic movement during breathing and consequently limiting diaphragmatic excursion. Additionally, the heart and abdominal organs might have shifted toward the load-bearing side, further affecting diaphragmatic motion (21). Moreover, fatigue from the imaging procedure, during which semi-prone measurements followed supine measurements, might have also affected the results.

Thoracic expansion in the R-L direction was slightly greater in the semi-prone position than in the supine position, and in the A-P direction, the left hemithorax expanded more than the right. A possible explanation for these findings may be that, in the semi-prone position with the right side down, the H-F expansion of the right hemithorax is restricted by body weight, whereas the left hemithorax is relatively free from such loading. Additionally, anterior displacement of the heart in this position may further facilitate compensatory expansion of the left hemithorax in the R-L and A-P directions. However, the semi-prone position tends to induce axial rotation or torsion of the body; thus, measurements along single representative planes may not correspond exactly to the same anatomical cross-sections as in the supine position. Future studies should incorporate area-based comparisons and further extend the methodology to 3D imaging along with more comprehensive volume-based assessments.

The reproducibility analysis demonstrated that intra- and interobserver agreement was generally high, particularly for measurements other than the R-L expansion. This robustness can be attributed to the use of green contour overlays, which helped clearly identify thoracic boundaries even in low spatial resolution images, thereby minimizing observer-dependent variability. In contrast, the R-L direction, which exhibited relatively low ICCs, likely reflects the small magnitude of thoracic expansion (typically only a few millimeters) in that direction, making it inherently more susceptible to minor measurement deviations. For test-retest reproducibility, although ICCs were overall lower than those for intraobserver evaluation, they still exceeded 0.8 in most directions, indicating good consistency across separate imaging sessions. This suggests that the combination of standardized participant's setup with a stable and precise inclination angle using positioning blocks, free of physical compression and positional constrains associated with the use of phased-array coils, and a reproducible analysis pipeline contributed to measurement stability, despite the sessions being conducted a week apart. Variations in physiological state, particularly in breathing condition, between sessions may have contributed to the observed decline in ICC values. Moreover, the extremely small sample size (*n* = 5) likely resulted in statistical instability, such as wide confidence intervals and occasional extreme ICC values. Thus, ICCs alone should not be overinterpreted, and reference should also be made to Supplementary Tables S1, S2, which present the detailed measurement data across observers and sessions. These findings underscore that, despite inherent limitations, the proposed method offers a reliable and reproducible framework for assessing respiratory thoracic motion.

Overall, the MR imaging and analysis method proposed in this study has proven highly effective in visualizing thoracic movements, with robust reproducibility in both 1D and 2D MR-based kymography. Future developments aimed at further automating kymogram analysis and improving contour visualization could lead to more stable and reliable measurements. Since it does not require any specialized phased-array coils for image acquisition and can be performed using freeware, such as ImageJ, kymography using our proposed method can be performed at a very low cost. Moreover, the presentation of the specific MR imaging protocols in this study, along with the wide accessibility of this method, improves the generalizability of our findings and enables broader clinical application of our proposed method. Although the achieved temporal resolution of 0.6 s may not always be sufficient to precisely capture the exact moments of end-inspiration and end-expiration, obtaining measurements from 60 s respiratory time series likely enabled these critical moments to be captured in at least one of the cycles. Furthermore, this method allows for free breathing, making it possible to increase the number of evaluated respiratory durations if needed. Combining this approach with EIT, which offers extremely high temporal resolution, could allow for an integrated assessment that leverages the spatial accuracy of MR-based morphological evaluation and the temporal precision of EIT-based ventilation analysis, as recently demonstrated during prone ventilation in patients with acute respiratory distress syndrome (29). Applying this MR–EIT integration in both prone and semi-prone configurations may help clinicians fine-tune positional strategies and optimize respiratory management for individual patients.

This study has several limitations that should be acknowledged. Most importantly, this was a proof-of-concept study with a very small sample size (*n* = 5) designed to establish the technical feasibility of body coil-only MR imaging for thoracic motion visualization rather than to provide precise quantitative measurements. The quantitative values for thoracic expansion and reproducibility metrics should be interpreted as preliminary technical benchmarks demonstrating methodological feasibility, rather than as definitive physiological parameters. Furthermore, the accuracy and precision of measurements at this stage are inherently limited by simplified analytical techniques, temporal and spatial resolution constraints, and the prioritization of SNR under body coil-only imaging. Additional limitations include the restriction to healthy males, which, combined with the small sample size, limits generalizability given reported sex differences in diaphragmatic movement (30). We did not assess clinically significant parameters such as actual ventilation or pulmonary blood flow, which would be essential for clinical applications in patients with respiratory diseases. Additionally, measurements were performed only in the central thoracic region, potentially missing regional variations in diaphragmatic motion due to its dome-shaped structure (27, 31). Furthermore, thoracic expansion was defined as changes along single axes within single imaging planes, which may limit accuracy of inter-positional comparisons due to inevitable body rotation or torsion across different positions. Despite these limitations, this study establishes a foundation for future investigations. Future studies should incorporate larger and more diverse samples, extend measurements to multiple thoracic regions and various positions (including lateral decubitus, prone, and left-sided variants) using area-based approaches with anatomically matched cross-sections and, ultimately, volumetric assessments. Additionally, incorporating ventilation measurements and evaluating the distribution of ventilation and blood flow using MR imaging (32, 33) could advance the application of this method to patients with respiratory diseases. Technical improvements should focus on both imaging aspects through faster acquisition methods and enhanced image contrast, and analytical aspects through refining the pipeline with improved anatomical matching, use of ImageJ macros for automated volumetric analysis, enhanced contour visualization, and combination with high-temporal-resolution modalities such as EIT to enhance clinical applicability.

In conclusion, we devised a novel MR imaging and analysis method that can depict thoracic respiratory dynamics while maintaining natural breathing patterns, eliminating the need for phased-array receiver coils or breath-holding. The resultant kymogram allows for visualization of the spatiotemporal dynamics of thoracic movements and measurement of thoracic expansion. Furthermore, we demonstrated that this method provides highly reproducible analyses of respiratory movements in both the supine and semi-prone positions.

## Data Availability

The datasets presented in this article are not publicly available because of the small scale of the study and the potential risk of identifying individual participants. Requests to access the datasets should be directed to Akira Ishikawa, ishikawa.a.kobe@gmail.com.

## Supplementary material

Supplementary Video S1 Magnetic resonance-based 2D kymogram in the supine position, visualizing respiratory dynamics in the coronal plane.

Supplementary Video S2 Magnetic resonance-based 2D kymogram of the right hemithorax in the supine position, visualizing respiratory dynamics in the sagittal plane.

Supplementary Video S3 Magnetic resonance-based 2D kymogram in the semi-prone position, visualizing respiratory dynamics in the coronal plane.

Supplementary Video S4 Magnetic resonance-based 2D kymogram of the right hemithorax in the semi-prone position, visualizing respiratory dynamics in the sagittal plane.
