# Supplementary material for: Novel magnetic resonance imaging methodology for dynamic visualization of respiratory thoracic motion: a pilot feasibility study
Source: Front Rehabil Sci. 2025 Aug 12;6:1540183. doi: 10.3389/fresc.2025.1540183 (PMC12378630; doi:10.3389/fresc.2025.1540183)
Supplement: Supplementary file 5 [file Table1.docx]

| **Table S1.** Thoracic dimensions at end-inspiration and end-expiration, and their expansion in the supine position | | | | | | | | | | | | |
| --- | --- | --- | --- | --- | --- | --- | --- | --- | --- | --- | --- | --- |
| Measurement axes | First imaging session | | | | | | | | | Second imaging session | | |
|  | Observer 1 | | | | | | Observer 2  (Single analysis session) | | | Observer 1  (Single analysis session) | | |
|  | First analysis session | | | Second analysis session | | |  |  |  |  |  |  |
|  | End-Insp  (mm) | End-Exp  (mm) | Expan-sion (mm) | End-Insp  (mm) | End-Exp  (mm) | Expan-sion (mm) | End-Insp  (mm) | End-Exp  (mm) | Expan-sion (mm) | End-Insp  (mm) | End-Exp  (mm) | Expan-sion (mm) |
| Right H-F | 216±19 | 129±21 | 87±21 | 216±20 | 130±21 | 86±22 | 216±20 | 128±21 | 88±21 | 216±22 | 134±24 | 82±21 |
| Left H-F | 222±17 | 141±14 | 81±17 | 219±15 | 139±14 | 81±17 | 222±16 | 140±15 | 81±15 | 218±11 | 136±19 | 81±21 |
| Bilateral R-L | 260±13 | 256±13 | 4±1 | 260±13 | 256±13 | 4±2 | 261±14 | 256±13 | 5±2 | 262±13 | 258±14 | 4±1 |
| Right A-P | 140±18 | 124±13 | 15±13 | 139±18 | 124±12 | 15±13 | 140±19 | 124±12 | 16±14 | 140±17 | 128±14 | 12±7 |
| Left A-P | 140±9 | 125±12 | 15±12 | 140±9 | 125±12 | 14±12 | 141±9 | 125±13 | 16±11 | 141±9 | 128±10 | 13±9 |

Data are presented as mean ± standard deviation. H-F: head–foot; R-L: right–left; A-P: anterior–posterior, End-Insp: end-inspiration, End-Exp: end-expiration. Expansion = End-Insp – End-Exp.

| **Table S2.** Thoracic dimensions at end-inspiration and end-expiration, and their expansion in the semi-prone position | | | | | | | | | | | | |
| --- | --- | --- | --- | --- | --- | --- | --- | --- | --- | --- | --- | --- |
| Measurement axes | First imaging session | | | | | | | | | Second imaging session | | |
|  | Observer 1 | | | | | | Observer 2  (Single analysis session) | | | Observer 1  (Single analysis session) | | |
|  | First analysis session | | | Second analysis session | | |  |  |  |  |  |  |
|  | End-Insp  (mm) | End-Exp  (mm) | Expan-sion (mm) | End-Insp  (mm) | End-Exp  (mm) | Expan-sion (mm) | End-Insp  (mm) | End-Exp  (mm) | Expan-sion (mm) | End-Insp  (mm) | End-Exp  (mm) | Expan-sion (mm) |
| Right H-F | 189±22 | 124±22 | 65±22 | 190±21 | 124±21 | 66±22 | 190±22 | 124±22 | 67±23 | 193±25 | 126±27 | 67±21 |
| Left H-F | 228±14 | 157±21 | 71±20 | 229±14 | 158±21 | 71±20 | 229±13 | 157±21 | 72±20 | 231±16 | 165±16 | 66±12 |
| Bilateral R-L | 261±18 | 252±15 | 8±4 | 260±17 | 252±14 | 8±4 | 261±19 | 251±15 | 10±6 | 258±19 | 250±15 | 9±5 |
| Right A-P | 133±22 | 125±18 | 9±5 | 133±21 | 124±17 | 9±6 | 133±22 | 124±19 | 10±4 | 136±16 | 127±15 | 9±4 |
| Left A-P | 144±14 | 128±13 | 17±11 | 144±13 | 128±12 | 16±10 | 145±14 | 128±14 | 17±13 | 142±16 | 127±15 | 15±9 |

Data are presented as mean ± standard deviation. H-F: head–foot; R-L: right–left; A-P: anterior–posterior, End-Insp: end-inspiration, End-Exp: end-expiration. Expansion = End-Insp – End-Exp.
